# Amino acid encoding for deep learning applications

**DOI:** 10.1186/s12859-020-03546-x

**Published:** 2020-06-09

**Authors:** Hesham ElAbd, Yana Bromberg, Adrienne Hoarfrost, Tobias Lenz, Andre Franke, Mareike Wendorff

**Affiliations:** 1 Institute of Clinical Molecular Biology, Christian-Albrechts-University of Kiel, Kiel, Germany; 2 Department of Biochemistry and Microbiology, Rutgers University, New Brunswick, NJ USA; 3 Department of Genetics, Rutgers University, New Brunswick, NJ USA; 4 Technical University of Munich Institute for Advanced Study, (TUM-IAS), Lichtenbergstr. 2a, 85748 Garching/Munich, Germany; 5 Research Group for Evolutionary Immunogenomics, Max Planck Institute for Evolutionary Biology, 24306 Plön, Germany

**Keywords:** Deep-learning, Amino acid encoding, Amino acids embedding, Protein-protein interaction (PPI), HLA-II peptide interaction, Convoluted-neural network (CNN), Recurrent neural network (RNN), Machine-learning (ML), Human-leukocyte antigen (HLA)

## Abstract

**Background:**

The number of applications of deep learning algorithms in bioinformatics is increasing as they usually achieve superior performance over classical approaches, especially, when bigger training datasets are available. In deep learning applications, discrete data, e.g. words or n-grams in language, or amino acids or nucleotides in bioinformatics, are generally represented as a continuous vector through an embedding matrix. Recently, learning this embedding matrix directly from the data as part of the continuous iteration of the model to optimize the target prediction – a process called ‘end-to-end learning’ – has led to state-of-the-art results in many fields. Although usage of embeddings is well described in the bioinformatics literature, the potential of end-to-end learning for single amino acids, as compared to more classical manually-curated encoding strategies, has not been systematically addressed. To this end, we compared classical encoding matrices, namely one-hot, VHSE8 and BLOSUM62, to end-to-end learning of amino acid embeddings for two different prediction tasks using three widely used architectures, namely recurrent neural networks (RNN), convolutional neural networks (CNN), and the hybrid CNN-RNN.

**Results:**

By using different deep learning architectures, we show that end-to-end learning is on par with classical encodings for embeddings of the same dimension even when limited training data is available, and might allow for a reduction in the embedding dimension without performance loss, which is critical when deploying the models to devices with limited computational capacities. We found that the embedding dimension is a major factor in controlling the model performance. Surprisingly, we observed that deep learning models are capable of learning from random vectors of appropriate dimension.

**Conclusion:**

Our study shows that end-to-end learning is a flexible and powerful method for amino acid encoding. Further, due to the flexibility of deep learning systems, amino acid encoding schemes should be benchmarked against random vectors of the same dimension to disentangle the information content provided by the encoding scheme from the distinguishability effect provided by the scheme.

## Background

Deep learning has recently received a lot of attention due to the major breakthroughs it has enabled in computer vision, machine translation, and bioinformatics. In bioinformatics, deep learning has been applied, for example, to SNP and small indel calling [1], to estimate the impact of non-coding variants on DNA-methylation [2], as well as for the prediction of protein function [3], structure [4], and protein-protein interactions (PPI) [5].

A critical step before feeding amino acid sequences to the model is numerical encoding through an encoding scheme that assigns a numerical representation to each amino acid, i.e. it is a map from the input amino acids to some point in the representational space of the scheme. Arguably, any encoding scheme should fulfill two requirements; First *distinguability*, meaning that you are able to distinguish or discriminate between the elements that the scheme is supposed to encode, e.g. amino acids; Second, *preservability*, meaning that the scheme is capturing or preserving the relationship among the elements of the scheme, usually this relationship is expressed geometrically through the vector representation of the encoded elements. Benchmarking of different amino acid encoding schemes for predicting amino acid similarity demonstrated that the encoding process plays a critical role in the applicability and quality of the model [6].

Despite the importance of biological sequence encoding, however, the investigation and development of encoding schemes for biological sequence information have not caught up with the accelerated development of deep learning models. Most of the newly developed models still use encoding schemes that were developed in the pre-deep-learning era. For example, orthogonal encoding, commonly known as “one-hot encoding” [7], substitution matrices such as the BLOck SUbstitution Matrix (BLOSUM) [8], and physicochemical character-based schemes such as the principal components score Vectors of Hydrophobic, Steric, and Electronic properties (VHSE8) [9], are commonly employed.

Notably, while providing a numerical representation of amino acid sequences, these encoding schemes often try to capture prior domain knowledge about amino acids as similarities between vectors. Where one-hot encoding assumes no prior knowledge, BLOSUM captures evolutionary relationships, and VHSE8 captures physicochemical properties. However, most of the current encoding schemes, which depend upon manually curated features, are general-purpose encoding schemes that are not optimized for the specific tasks at hand [10]. In addition, manually curated encoding schemes are dependent on current domain knowledge which may not capture features that are important for governing the relationships among amino acid sequences that are not yet known.

Recently, machine learning has been applied to biological sequences, taking advantage of the expanding sequence repositories to learn a meaningful numerical representation for biological sequences. For example, Asgahri and Mofrad [11] used unsupervised learning in a fashion similar to word2vec [12] to develop ProtVec which is a one-hundred dimension learned vector representation for fixed overlapping k-mers of amino acids. More recently, Rives and colleagues [13] used bidirectional contextual language models for learning a representation for whole protein sequences while Alley and colleagues used an LSTM-based architecture to learn an embedding of whole protein sequences into a fixed-length vector [14]. This form of unsupervised learning is usually used as a starting point for training task-specific models, i.e. pre-training for a supervised learning task.

Another possible way for representing input sequences in a task-specific manner is by making the encoding a learnable part of the model, i.e. by jointly learning the encoding scheme with other model parameters (here, end-to-end learning). Arguably, end-to-end learning has been particularly effective as data availability increases and deep learning models can be trained on very large datasets. In such data-intensive cases, the models may be able to capture features which underlie similarities and differences between amino acids which are not captured by classical manually-curated encodings. It might also enable the model to encode, learn and extract the aspects of amino acids that are relevant for the task-at-hand which might differ between different tasks. Such applications may require a minimum amount of training data, and the threshold for this data size in bioinformatics remains unconstrained.

Recently, Raimondi and colleagues [15], have performed an in-depth analysis of amino acid encoding using biophysical propensity scales for shallow machine-learning models where they argued that a learned embedding of features might lead to a simple, optimal and assumptions-free feature engineering. Nevertheless, to the best of our knowledge, in peptidomics and proteomics there has not been a formal comparison between classical encoding and end-to-end learning, especially, for deep-learning model. Therefore, we here aimed to evaluate the performance of different classical single amino-acid encoding schemes, focusing on one-hot, VHSE8 and BLOSUM62, as compared to end-to-end learning. We evaluate the performance of end-to-end learning relative to classical encoding schemes across different data sizes. To disentangle the information content in the encoding scheme as represented by the geometrical relationship among its vectors from the distinguishability effect provided by the unique position of each amino acid in the embedding space, we compare learned and classical encoding strategies to ‘random frozen embedding’ of amino acids, i.e. by randomly assigning a unique position to each amino acid in the embedding space. Finally, we examine the consistency of the effect of learned, classical, and random frozen embedding on model performance across different model architectures and two challenging biological problems, predicting human leukocyte antigen class II (HLA-II)-peptide interactions and PPIs.

## Results

### End-to-end learning shows comparable performance to different classical encoding schemes but at a lower embedding dimension

**Fig. 1:**
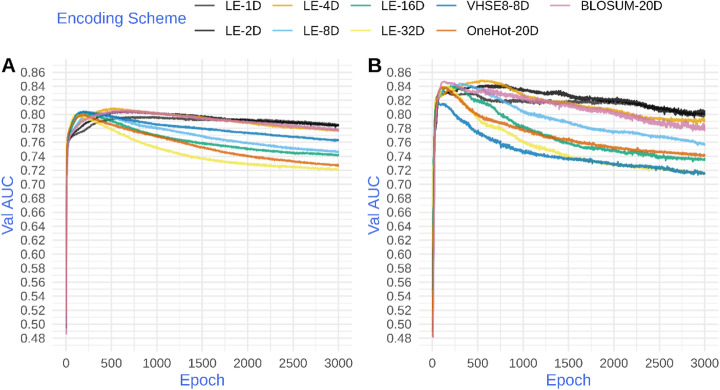
*Comparison between classical and machine-learnt (LE) encoding schemes for LSTM-based models of peptide-HLA-II interaction*. The y-axis shows the area under the receiver operating characteristic curve, AUC, for the model predictions on the validation dataset (Val AUC). The x-axis shows the number of training cycles or epochs. **a** shows the performances of models trained on HLA-DRB1*15:01 data and **b** shows the model performances for HLA-DRB1*13:01 data

We first compared the performance of machine-learnt encoding schemes, i.e. end-to-end learning, to classical encoding schemes. In brief, for the machine-learnt encoding, an embedding layer was used with one, two, four, eight, sixteen or thirty-two as the embedding dimension while for classical encoding the weights of the embedding layer were replaced with the encoding matrix and those weights were kept fixed during training. We then trained long short-term memory (LSTM) based models for predicting the affinity of peptides toward one of two HLA-II proteins namely, HLA-DRB1*15:01 and HLA-DRB1*13:01 (see Methods). As shown in Fig. 1a and b, end-to-end learning allows the model to achieve the same performance as classical encoding but using a lower embedding dimension. For example, a 4-dimensional learned embedding (LE) achieved comparable performance compared to a 20-dimensional classical encoding (see LE-4D compared to BLOSUM62-20D and OneHot20D, Fig. 1a and b). High dimensional encoding schemes, regardless of being machine-learnt or classical, showed a higher degree of overfitting over longer training times, resulting in a lower validation AUC. However, different encoding schemes achieved similar maximum performance in early epochs regardless of the embedding dimension. To test if these results are stable across different architectures, we constructed CNN-LSTM based models (see Methods) and trained these models on the peptide-HLA-II data. Similar to the LSTM-based model architecture, a CNN-LSTM model achieved superior or comparable performance using end-to-end learning compared to classical encodings, and this performance was achieved using a lower embedding dimension than the classical encodings (Fig. S1 A and B).

### End-to-end learning consistently enables efficient encoding of amino acids across different problems, different architectures and different amount of training data

**Fig. 2:**
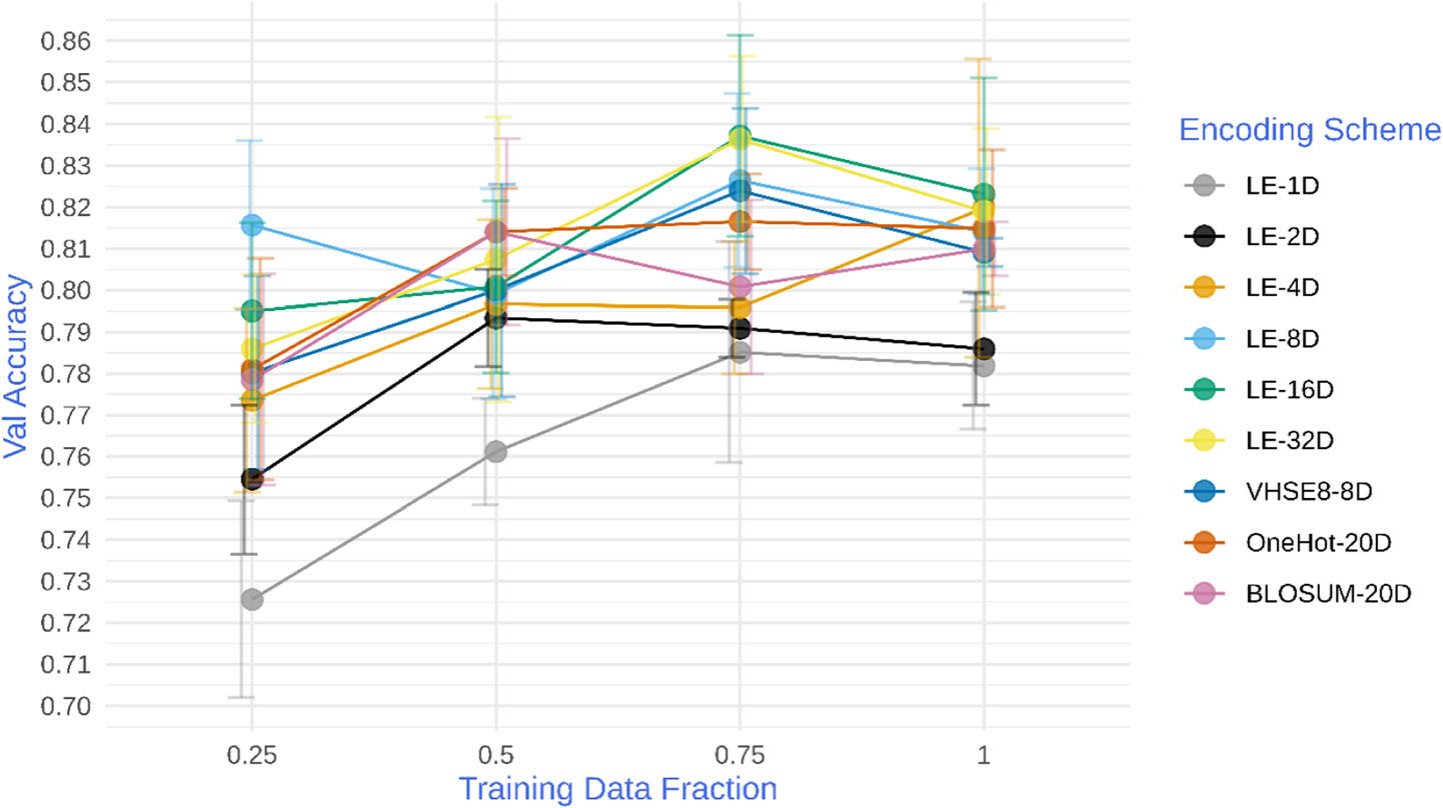
*Comparison between classical and machine-learnt (LE) encoding schemes for PPI-based models.* The y-axis shows the accuracy of the model prediction on the validation dataset while the x-axis shows the fraction of the training dataset used to train the models. Error-bars represent one standard-deviation from the mean of five repetitions

Next, we were interested in seeing if end-to-end learning will show the same patterns in performance across different architectures, prediction tasks, and data sizes. To this end, we compared end-to-end learned embeddings to classical embeddings on a new task with a different model architecture: predicting PPIs from amino acid sequence information, using a model architecture adapted from Hashemifar and colleagues [5]. Our selected model was a smaller version of the previously published model [5] to enable rapid experimentation (see Methods). To test the effect of training data size on embedding performance, the model was also trained with different subsets of the training data, restricting data inputs to 25, 50, 75 and 100% of the total dataset, respectively. As shown in Fig. 2, end-to-end learning enables the model to achieve a performance that exceeds all classical encoding schemes in almost all cases at 25, 75, and 100% data fractions, and exceeded or met all but the BLOSUM encoding at 50%. As data size increased, the improvement of end-to-end encoding over classical encoding schemes was more pronounced, and was able to exceed the performance of classical encodings with far fewer embedding dimensions (Fig. 2).

### The learnt embedding space captured meaningful aspects of amino acids physicochemical properties

**Fig. 3:**
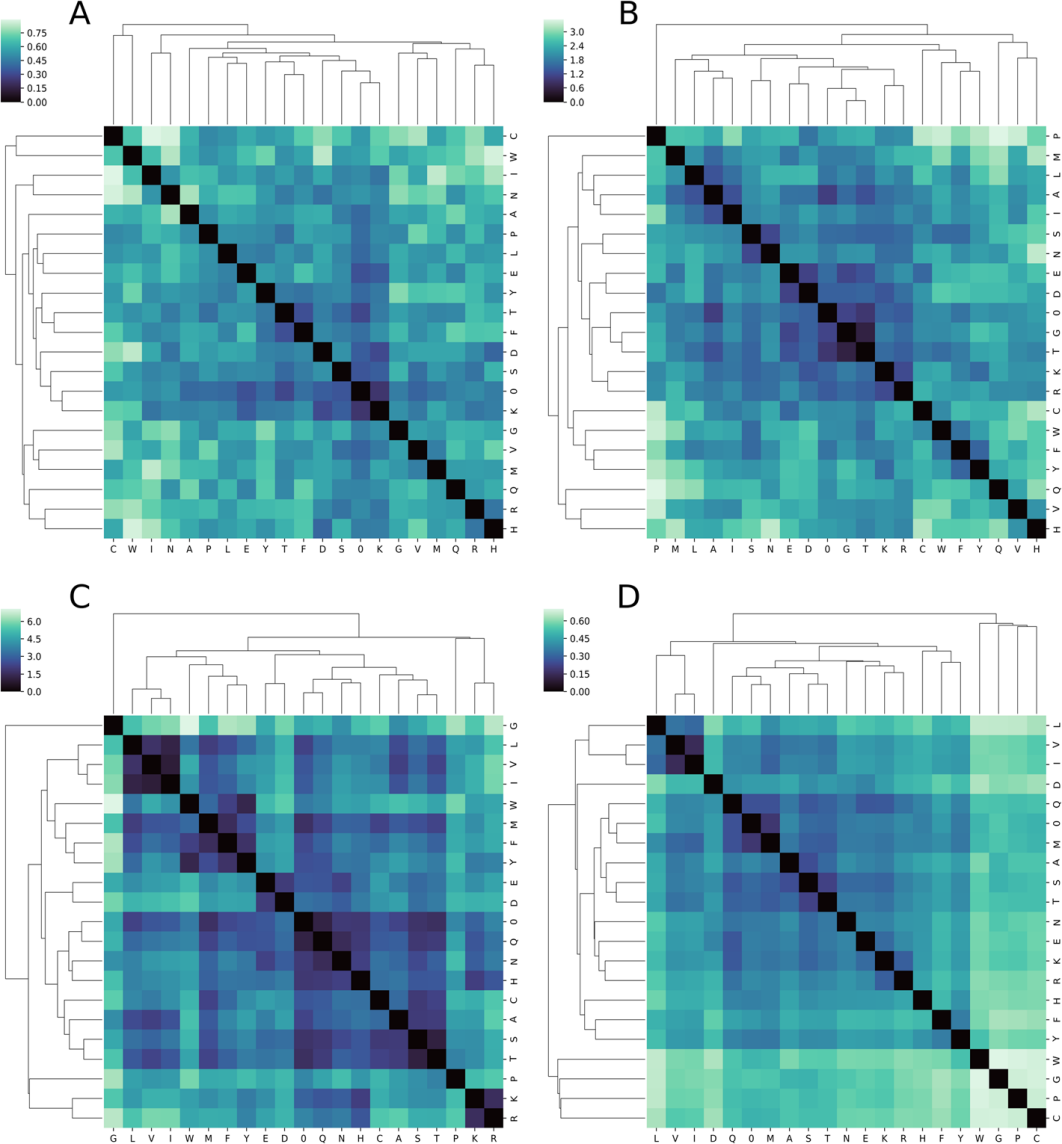
*Cluster heat-map of the pairwise distance among amino acids in different encoding schemes.***a** represents the learnt embedding space of a PPI-based model with eight dimensions. **b** represents the learnt embedding space of a peptide-HLA-II LSTM-based model with eight dimensions. **c** represents VHSE8 Matrix. **d** represents BLOSUM62. For the PPI-based model, the model was trained for 50 epochs using the full training dataset as described in the Materials and Methods section. For the RNN-based model, the model was trained for 3000 epochs using HLA-DRB1*15:01 data as described in Materials and Methods. For both cases, the weights of the embedding layer after training was used for visualizing the learnt embedding space. The special character zero, used for padding shorter sequences, was also included in the analysis

To get a better understanding of information captured by the learnt embedding space as compared to the classical encoding schemes, we compared the Euclidean distance among the vector representations of each amino acid embedding under four encoding schemes: the 8-dimensional learnt embedding of PPI-based models (Fig. 3a); the 8-dimensional learnt embedding of RNN-based models (Fig. 3b); and the physicochemical based encoding schemes of VHSE-8 (Fig. 3c) and BLOSUM (Fig. 3d). The learnt embedding space (Fig. 3a and b) do not produce as distinct of clusters along physicochemical lines as those observed with VHSE-8 (Fig. 3c) and to some extent BLOSUM (Fig. 3d). Nevertheless, there is some partitioning in the learnt embedding space along physicochemical lines: as shown in Fig. 3b, the amino acids with an aromatic side-chain – phenylalanine (F), tyrosine (Y) and tryptophan (W) – are located closer to each other in the learnt embedding space of the LSTM-based model for peptide-HLA-DRB1*15:01 interactions, as are amino acids with a hydrophobic side-chain – leucine (L), isoleucine (I), alanine (A), and methionine (M). The same clustering is also observed with the two amino acids with an acidic side-chain, aspartic acid (D) and glutamic acid (E), and with the two amino acid with a neutral side-chain, asparagine (N) and serine (S). Finally, arginine (R) and lysine (K), the two amino acids with a basic side-chain, are also located near each other in the embedding space. This suggests that the model was able to discover the physicochemical similarities among amino acid groups indirectly from the training dataset as it tries to find a pattern in the data that would minimize the loss on the task at hand. Importantly, this model may also be learning relationships among amino acids that are not defined by known physicochemical parameters, whereas manually curated classical encodings are limited by domain knowledge of the physicochemical similarity among amino acids. Interestingly, the embedding space of the PPI-based models seems to capture less knowledge about the physicochemical properties of amino acids. This difference in the embedding space between the learnt embeddings may be due to differences in learning styles of model architectures (see Discussion).

### Deep learning models are capable of learning from random vectors of appropriate dimensionality

**Fig. 4:**
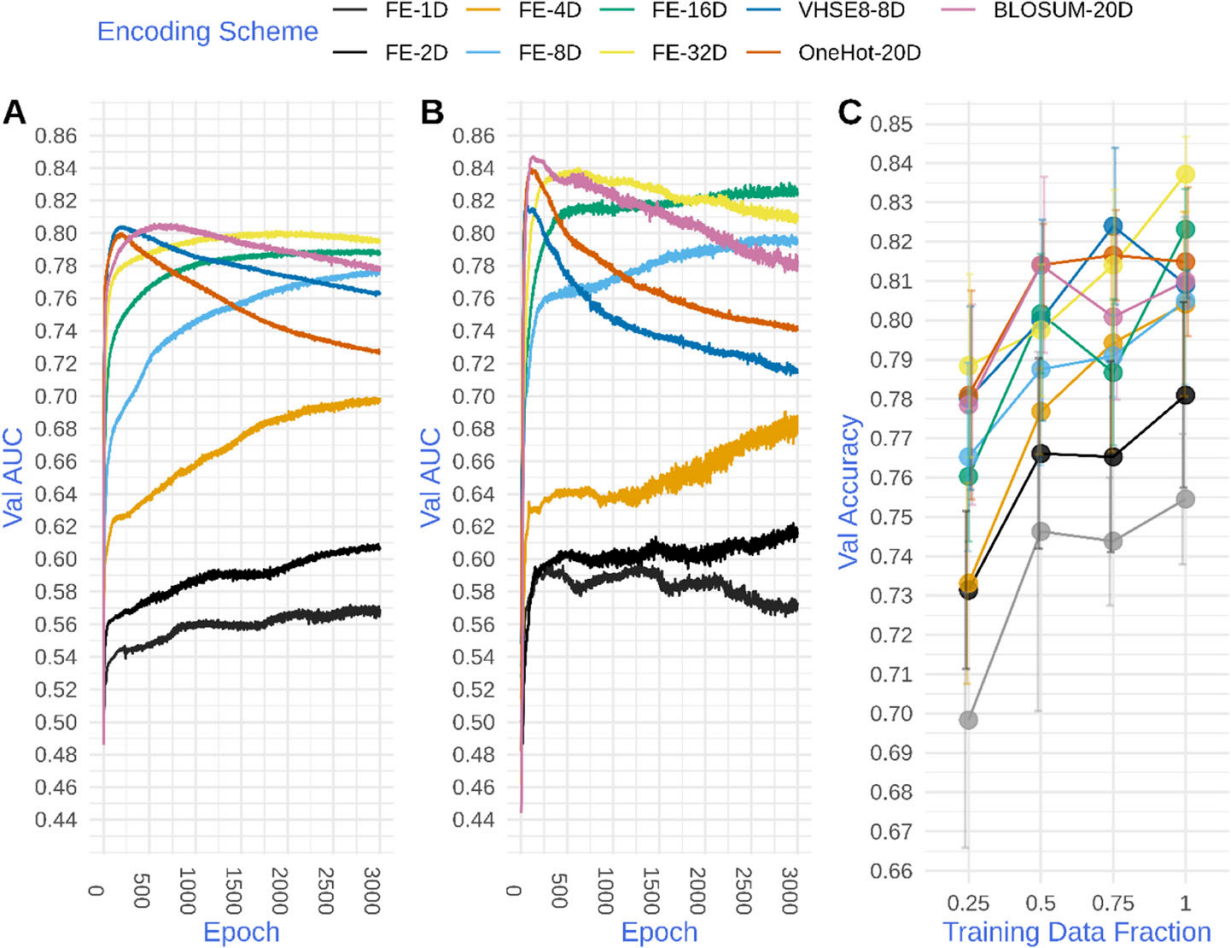
A comparison between classical encoding and random frozen embedding (FE). **a** shows the performances of models trained on HLA-DRB1*15:01 data and **b** shows the model performances for HLA-DRB1*13:01 data. The y-axis of **a** & **b** shows the area under the receiver operating characteristic curve, AUC, for the model predictions on the validation dataset (Val AUC), while, the x-axis shows the number of training cycles or epochs. **c** is the performance of PPI model trained on different fractions of the training data. The y-axis shows the accuracy on the validation dataset while the x-axis shows the fraction of training data used while error bars represent one standard-deviation from the mean of five repetitions

To disentangle the information content in the encoding scheme, which is encoded in the form of a geometrical relationship among the vector representation of amino acids from the distinguishability effect provided by the unique position of each amino acid in the space we used the same settings mentioned above, however, we prevented the update of the embedding weights during training. Hence, each amino acid was represented as a vector of random numbers drawn from a uniform distribution. Effectively, assigning each amino acid to a unique position in the embedding space, however, the relationship among these positions is completely arbitrary. As shown in Fig. 4a and b, in a low embedding dimension, for example, one or two dimensional space the models for peptide-HLA-II interaction over fit the training data and are not able to generalize to the un-seen examples in the validation dataset. However, as the dimensionality of the embedding space increases, the model performance on the validation dataset improves, and for higher numbers of dimensions, it achieved a comparable performance to both learned and classical encoding schemes.

This result is consistent across model architectures (Figs. S2 A and B) – CNN-LSTM based models trained using random frozen embeddings exhibited the same pattern as the LSTM-based models, i.e. the model performed poorly with few embedding dimensions and improved with increasing dimensionality, achieving comparable performance to classical and learned embeddings at the highest dimensions. Also, it was consistent across problems and across different amount of training data. As seen in Fig. 4c, the PPI model was able to learn from random vectors, and its ability to learn improved with increasing the dimensionality of the embedding space. This consistency suggests that the ability of the model to learn from random vectors of appropriate dimension is independent of the model architecture used, possibly because the model treats these random vectors in a “one-hot” fashion, i.e. it just uses the encoding scheme to distinguish between amino acids and shifts the information content, i.e. the relationship among the amino acids, to the next layer/layers of the model.

## Discussion

Our results demonstrate that end-to-end training achieves superior or comparable performance to classical encoding schemes, and may be the preferred approach to encoding of amino acid sequences under many modelling circumstances. End-to-end training can be used to achieve comparable performance to classical encoding schemes in a much lower embedding dimension, and achieves increasingly improved performance at higher embedding dimensions. This performance is consistent across three different neural architectures and two challenging bioinformatic problems, namely, predicting HLA-II-peptide interactions and PPIs. Performance improvements from end-to-end learning over classical encodings are particularly pronounced as data sizes increase, suggesting that as labelled data becomes more available or pre-trained embeddings from unsupervised approaches become more widely adopted, end-to-end learning is likely to be preferable to classical encodings. Even at the relatively restricted data sizes used in this study – for example, with modelling of peptide-HLA-DRB1*13:01 interactions fewer than 850 training data points were used – end-to-end learning achieved comparable or superior performance to classical encodings.

For applications where the trained model will be deployed to devices with limited computational and/or memory capacity, the ability to encode amino acids at a lower dimension than classical encodings becomes critical. For example, representing a sequence of 100 amino acids using BLOSUM will generate a 100*x*20 matrix, while embedding the same sequence in a learned embedding space of two or four would generate a 100*x*2 and 100*x*4 matrix, respectively. Using such a learned embedding thus reduces the encoded sequence size by 10 and 5 folds, respectively.

End-to-end learning offers many advantages in comparison to classical encoding schemes. For example, end-to-end learning can easily encode non-proteinogenic amino acids or post-translational modifications, when data is available, by simply increasing the vocabulary size and letting the machine learn how to encode it on its own, whereas manually curated classical encodings cannot easily be expanded in this straightforward manner. A second advantage for end-to-end learning is the straightforward ability to control the embedding dimension, which is an important factor in more advanced architectures like multi-headed attention [16] where the embedding dimension must be divisible by the number of attention heads.

We identified the embedding dimensionality as a major player in controlling the model performance. This result agrees with what has been reported before in the bioinformatics literature. For example, Liu and colleagues [17] reported that their model achieved the best performance when amino acids were encoded as vectors of size 43, containing a permutation of physical properties, BLOSUM, and one-hot encoding. Hence, our results argue that the encoding dimensionality should be treated as a critical hyperparameter that should be tuned in a task-specific manner.

We also detected that by simply embedding the amino acids randomly in a high-dimensional space, we achieved comparable performance to both the machine-learnt and classical amino acid encoding schemes. This observation was consistent and independent of the prediction task and the architecture used. The ability of machine-learning models to learn from random vectors has been previously described in the bioinformatics literature by Raimondi and colleagues [15] for shallow machine-learning models, and in the natural language processing literature by Kocmi and Bojar [18] for deep learning model. A key insight from these studies is the flexibility of non-linear machine-learning models, for example, Kocmi and Bojar [18] argued that when the network is faced with frozen embeddings it tries to learn in a “one-hot” fashion, i.e. it mainly depends upon the distinguishability among different tokens. In contrast to word embedding, where a vocabulary of tens of thousands of words are commonly used, here we used single amino-acid based encoding with a vocabulary size of twenty. Thus, distinguishing between different amino acids is relatively easy with increasing embedding dimension, particularly as the embedding dimension meets or exceeds the vocabulary size. This might explain the relationship between the dimensionality of the random frozen embeddings and the model performance, which improves with increasing dimensionality of the random frozen embeddings. At low embedding dimensions much smaller than the vocabulary size, the network struggles to distinguish between different amino acids, whereas at higher embedding dimensions similar to or exceeding the vocabulary size, the model performs comparably with models using one-hot encoded amino acids (Fig. 4). Hence, our findings argue that amino-acid encoding schemes should be benchmarked against random vectors of the same dimension to disentangle the information contained in the encoding scheme from the distinguishability effect that is provided by the dimension of the encoding scheme.

Interestingly, we found that the information content in the learned embedding space, as represented by the geometrical relationship between the amino acids, differs a lot between tasks. For example, the embedding space of the peptide-HLA-II models seems to capture more knowledge about the physio-chemical properties of amino acids in comparison to PPI-based models. This difference in embedding space between the learnt embeddings may be due to the difference in model architecture used, i.e. CNN vs. RNN. The CNN-based architecture tries to find translational-invariants in the input by convolving a different number of filters over a block of amino acids which differ from the way RNNs process their input sequences, which analyse one amino acid at a time. A second factor that might explain this difference, is the scale of both problems, wherein protein-protein interactions long protein sequences have been used, where the contribution of each amino acids is governed by other amino acids in its vicinity, hence, the context, or the high-level organization of blocks of amino acids into motifs, or even a higher level as domains, is more informative for the model than each amino acid individually. Hence, the lack of an organized embedding space for this problem. On the other hand, for HLA-II peptide interaction short peptides have been used, where a replacement of one amino-acid can completely change the binding between the peptide and the HLA-II protein. Hence, the models pay more attention to each individual amino acid and we get a much-more organized embedding space. Thus, we speculate that the difference in the embedding space between the two problems might be due to the nature of the problem itself and the employed underlying architectures.

## Conclusions

In this work, we compared end-to-end learning to feature-based classical encoding of amino acids. We compared the performance of these two encoding strategies using three commonly used neural architectures – recurrent neural networks (RNN), convolutional neural networks (CNN), and a hybrid CNN-RNN – as applied to two challenging problems – predicting human leukocyte antigen class II (HLA-II)-peptide interactions and protein-protein interactions (PPIs). As shown above, end-to-end learning allows for an efficient, scalable, and easy to fine-tune encoding without reducing performance. Nevertheless, given that each bioinformatics problem has a different performance requirement, different complexities and different amounts of data available, more experimentation would be highly needed to characterize the performance of end-to-end learning with regard to these problems.

We also find that the embedding dimensionality is an important hyperparameter that should be carefully tuned to fit the training set size and model complexity in order to improve the overall performance. Also, our experimentation with random embedding builds on what has been previously described by Raimondi and colleagues [15] where they showed that non-linear machine-learning models are not able to discriminate between real and random encoding schemes. Hence, we argue that newer encoding schemes should be benchmarked against frozen embeddings of the same dimension to disentangle the information content of the encoding scheme from the distinguishability effect provided by the encoding scheme.

## Methods

### Task definitions and data preparation

#### Peptide-HLA-II interaction dataset

HLA-II is a heteromeric protein that is predominantly expressed on antigen-presenting cells, where it presents peptides and protein fragments to CD4+ T cells [19]. Modelling the interaction between possible peptide candidates and HLA-II proteins is of paramount importance to understand the genetic association between certain alleles and autoimmune diseases, and to develop novel vaccines and immunotherapies. Here, we modeled the interaction between peptides and two HLA-II alleles of interest, HLA-DRB1*15:01 and HLA-DRB1*13:01, because of their genetic association with inflammatory bowel disease (IBD).

We used two publicly available datasets from *NetMHCIIpan* (http://www.cbs.dtu.dk/suppl/immunology/NetMHCIIpan-3.2/) [20] to train the peptide-HLA-II binding prediction models. In short, both datasets comprise pairs of peptides and alleles along with their log-transformed IC50 values as a measure of the binding affinity, for data from the two molecules HLA-DRB1*13:01 and HLA-DRB1*15:01. Each dataset was split into a five-fold cross-validation dataset by Jensen and colleagues [20], with about 800 training examples (HLA-DRB1*13:01) and ~ 3900 training examples (HLA-DRB1*15:01). Peptide sequences were processed by (i) encoding them as integers through a tokenizer, and (ii) padding shorter sequences and trimming longer peptide sequences from the head end to a fixed length of 26 amino acids. To compute the AUC, the IC50 values were binarized using the same threshold used by Jensen and colleagues of 0.426, which is equal to 500 nM [20].

#### Protein-protein interaction (PPI) dataset

The binary PPI data were downloaded from the HiNT database [21]. At the time of download (August 2019), the database contained PPI data for 12 organisms, namely, *H. sapiens, S. cerevisiae, S. pombe, M. musculus, D. melanogaster, C. elegans, A. thaliana, B. subtilis, B. taurus, E. coli, R. norvegicus and O. sativa.* The dataset was prepared for training as follows: (i) the binary interaction data, i.e. the positively interacting protein pair IDs for the 12 organisms were downloaded and combined, resulting in 163,165 interaction pairs across 44,340 unique proteins. (ii) The protein sequences were extracted where available from a local copy of the Swiss-Prot and Trembl databases [22]. The sequences of 43,084 proteins (97.167%) were successfully extracted from the databases. Next, proteins shorter than 100 amino acids or longer than 1000 amino acids were removed from the database. After filtration, the database contained 123,402 interacting pairs among 37,557 unique proteins.

The set of unique proteins were split into two sets, one for training containing 33,801 unique proteins (90%) and the second for testing containing 3756 unique proteins (10%). To make sure that the accuracy of the models on the test data was a result of a general pattern learned by the model and not due to homology between proteins in the test and training datasets, we removed any protein in the test dataset sharing more than 40% homology to any of the training set proteins. To remove homologous proteins overlapping in the test and training datasets, *blastp* [23] was used. In brief, the sequences of the proteins in the training dataset were used to construct a database and then the test proteins were blasted against it, and any test proteins with more than 40% homology were removed from the final test set. This procedure resulted in a test dataset with 591 unique proteins. Next, the binary interaction data for the proteins in the training and test dataset were extracted, resulting in a training dataset with 100,635 positively interacting pairs and a test dataset with 121 positively interacting pairs. To construct negative examples, random sampling was used to generate a dataset of equal size, i.e. the ratio of positive to negative examples is 1:1. The positive and negative pairs were combined to generate a training dataset with 201,270 examples and a test dataset with 242 examples. Finally, the protein sequences were encoded as integers through a tokenizer, and shorter sequences were zero-padded to a fixed length of 1000 amino acids.

### The embedding layer

The embedding layer is a look-up table or a weight matrix where each row is in our case a vector representation of a specific amino acid. The number of rows is equal to the number of unique vocabulary elements, i.e. number of amino acids, plus one, at the zero index, which is a reserved value for the padding variable. The number of columns is the embedding dimension, which is a model hyperparameter. Before training starts the weight matrix is initialized randomly along with all the parameters of the networks, and during the training phase the values inside the matrix are updated to minimize the error made by the network. The optimized embedding of each amino acid is thus “learned”from this iterative process of updating the weights of the embedding matrix. This differs from classical encoding where the numerical values for amino acids are not updated during training (Fig. S2).

If the model was trained using classical encoding, the weights of the layer were replaced with the encoding scheme and kept fixed during the training phase. In the case of BLOSUM62, a frequency normalized form was obtained from *NetMHCIIpan*3.2 software package [20] while for VHSE8 the raw values provided by the authors [9] have been directly used. Otherwise, all weights, i.e. the elements of the matrix, were drawn from a uniform distribution over the interval [− 0.05, + 0.05], which is the default initializer for the layer as implemented in Keras [24]. If random frozen encoding were used, these elements were kept fixed, i.e. they were not updated during the training phase. If learnt-encoding was used, the weights were adjusted using backpropagation to optimize the model objective function.

### Model architectures

#### An LSTM-based model for HLA-II peptide interaction prediction

The model is a composite of three layers. The first layer is an embedding layer which takes as an input an integer which maps the identity of the amino acid to the corresponding row index in the embedding matrix, and converts the amino acid index to a vector in the embedding space. The embedding layer is followed by a long short-term memory (LSTM) layer with 12 nodes, and the final layer consists of a single neuron producing the network output. As the normalized IC50 values ranged from zero to one, we applied a sigmoidal activation function to the output of the final layer to restrict the range of the model predictions to this range. The mean absolute error was used as a loss function and Adam [25] was used as an optimizer. The model was implemented using the Keras API [24] built on the TensorFlow deep learning framework [26]. Training the model was carried out in batches of size 256 using an Nvidia Tesla V100-SXM2 GPU.

#### A CNN-LSTM-based model for HLA-II peptide interactions

To evaluate the impact of the model architecture, we built another model composed of four components: an embedding layer, followed by a small convolution layer with 36 filters each of size nine and a stride size (or step size) of one, followed by an LSTM with 12 nodes, and finally a prediction neuron. Sigmoid was used as an activation function for the prediction units, the mean absolute error was used as a loss function and Adam [25] as an optimizer. The model was implemented using the Keras API [24] built on the TensorFlow deep learning framework [26]. Training the model was carried out in batches of size 256 examples using an Nvidia Tesla V100-SXM2 GPU.

#### A Siamese-like CNN for protein-protein interaction (PPI) prediction

Using TensorFlow, we implemented an architecture inspired by Hashemifar and colleagues [5] for identifying PPIs. This model is composed of two parts. The first is a convolutional neural network (CNN) for processing individual protein sequences. It is a composite of four modules, each of which contains a convolution layer, followed by a rectified linear unit (ReLu), a batch normalization layer, and an average pooling layer, except the final module which uses Global average pooling. Each module is implemented with a variable number of filters and kernel sizes (Table S1). The CNN at each forward pass receives as an input a pair of input proteins, and produces a pair of vectors that represent each of these proteins using the same weights; hence it is a Siamese-like architecture. The second part of the model is a feed-forward multilayer perceptron, which receive the vectors produced by the CNNs and returns the probability that the pair of proteins interact. Our reported model is considerably smaller than the one described by Hashemifar and colleagues [5]. For example, we used four convolution modules instead of five. The aim of this reduction in size was to allow faster training and experimentation with different dimensionality. A sigmoid function was used as an activation function for the prediction units, the binary cross-entropy was used as a loss function and Adam [25] as an optimizer. The model was implemented using the Keras API [24] built on the TensorFlow deep learning framework [26]. Training the model was carried out in batches of size 1024 using Nvidia Tesla V100-SXM2 GPUs.

## Supplementary information

**Additional file 1 Fig. S1**. Comparison between classical encoding and machine-learned (LE) encoding schemes used to encode amino acids for a CNN-LSTM based peptide-HLA-II interaction model. **Fig. S2**: Comparison between classical encoding and random frozen embedding (LE) encoding schemes used to encode amino acids for a CNN-LSTM based peptide-HLA-II interaction model. **Fig. S3**: Comparison between classical encoding schemes and machine-learnt encoding schemes. **Table S1**: The parameters of the four convolution modules used with the model. For convolutional module 1–3 average pooling was used and for convolutional module four global average pooling was used.

## Data Availability

The code and the datasets generated during the current study are available in the amino_acid_encoding_deep_learning_applications repository, https://github.com/ikmb/amino_acid_encoding_deep_learning_applications

## Abbreviations

BLOSUM: BLOck Substitution Matrix
CNN: Convolutional neural networks
HLA: Human leukocyte antigen
LSTM: Long short-term memory
SNP: Single nucleotide polymorphism
PPI: Protein-protein interaction
VHSE8: Physicochemical character-based schemes such as the principal components score Vectors of Hydrophobic, Steric, and Electronic properties
