# Supplementary material for: Amino acid encoding for deep learning applications
Source: BMC Bioinformatics. 2020 Jun 9;21:235. doi: 10.1186/s12859-020-03546-x (PMC7285590; doi:10.1186/s12859-020-03546-x)
Supplement: Supplementary file 1 — Additional file 1 Fig. S1. Comparison between classical encoding and machine-learned (LE) encoding schemes used to encode amino acids for a CNN-LSTM based peptide-HLA-II interaction model. Fig. S2: Comparison between classical encoding and random frozen embedding (LE) encoding schemes used to encode amino acids for a CNN-LSTM based peptide-HLA-II interaction model. Fig. S3: Comparison between classical encoding schemes and machine-learnt encoding schemes. Table S1: The parameters of the four convolution modules used with the model. For convolutional module 1–3 average pooling was used and for convolutional module four global average pooling was used. [file 12859_2020_3546_MOESM1_ESM.docx]

**Supplementary Figures:**


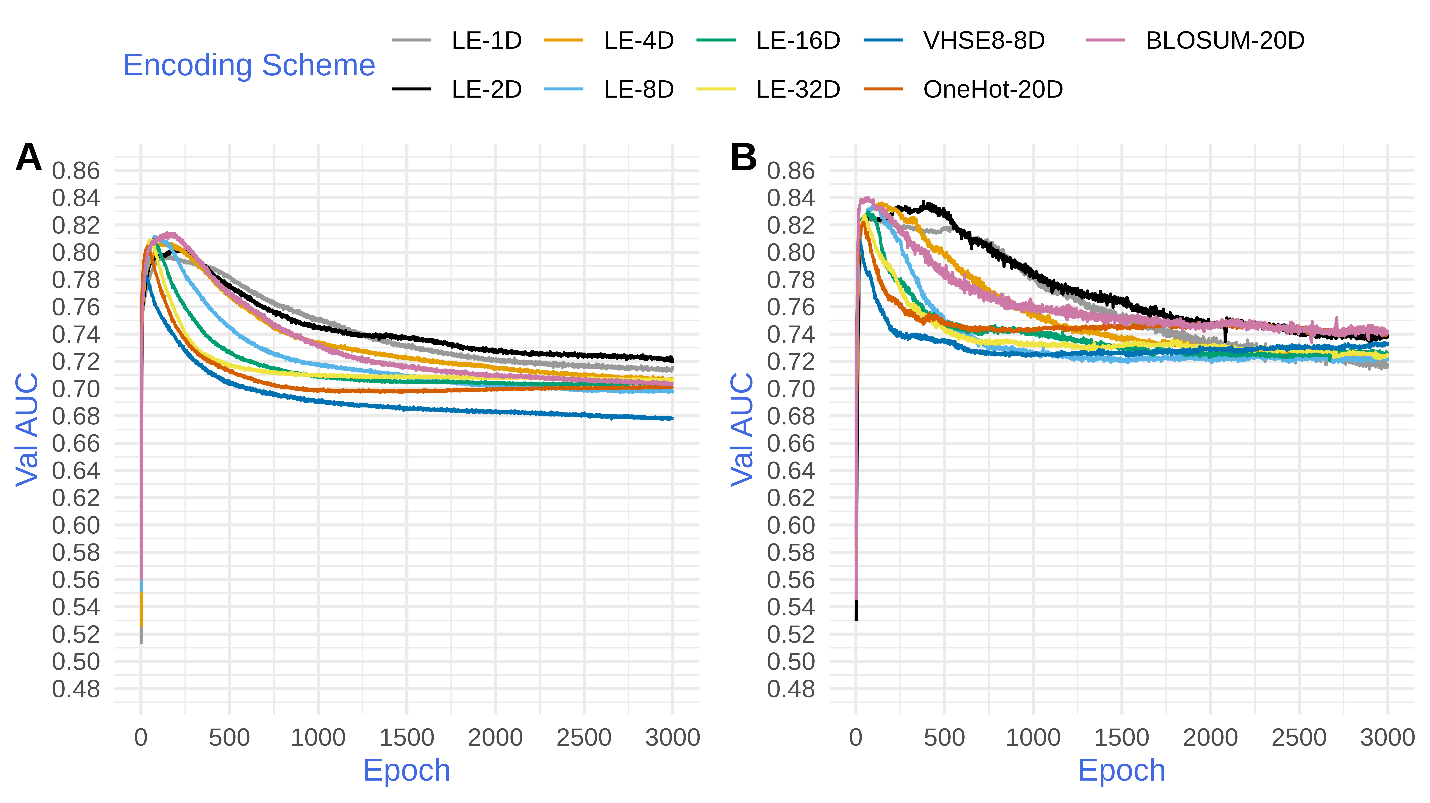


**Fig. S1:** Comparison between classical encoding and machine-learned (LE) encoding schemes used to encode amino acids for a CNN-LSTM based peptide-HLA-II interaction model. The y-axis shows the area under the receiver operating characteristic curve (ROC), AUC, for the model predictions on the validation dataset (Val AUC). The x-axis shows the number of training cycles or epochs. (A) shows the performances of models trained on HLA-DRB1*15:01 data and (B) shows the model performances for HLA-DRB1*13:01 data. As training deep learning models is a non-deterministic process, each line in the graph represents the average performance over three repetitions of a five-fold cross-validation dataset, i.e. fifteen different experiments.


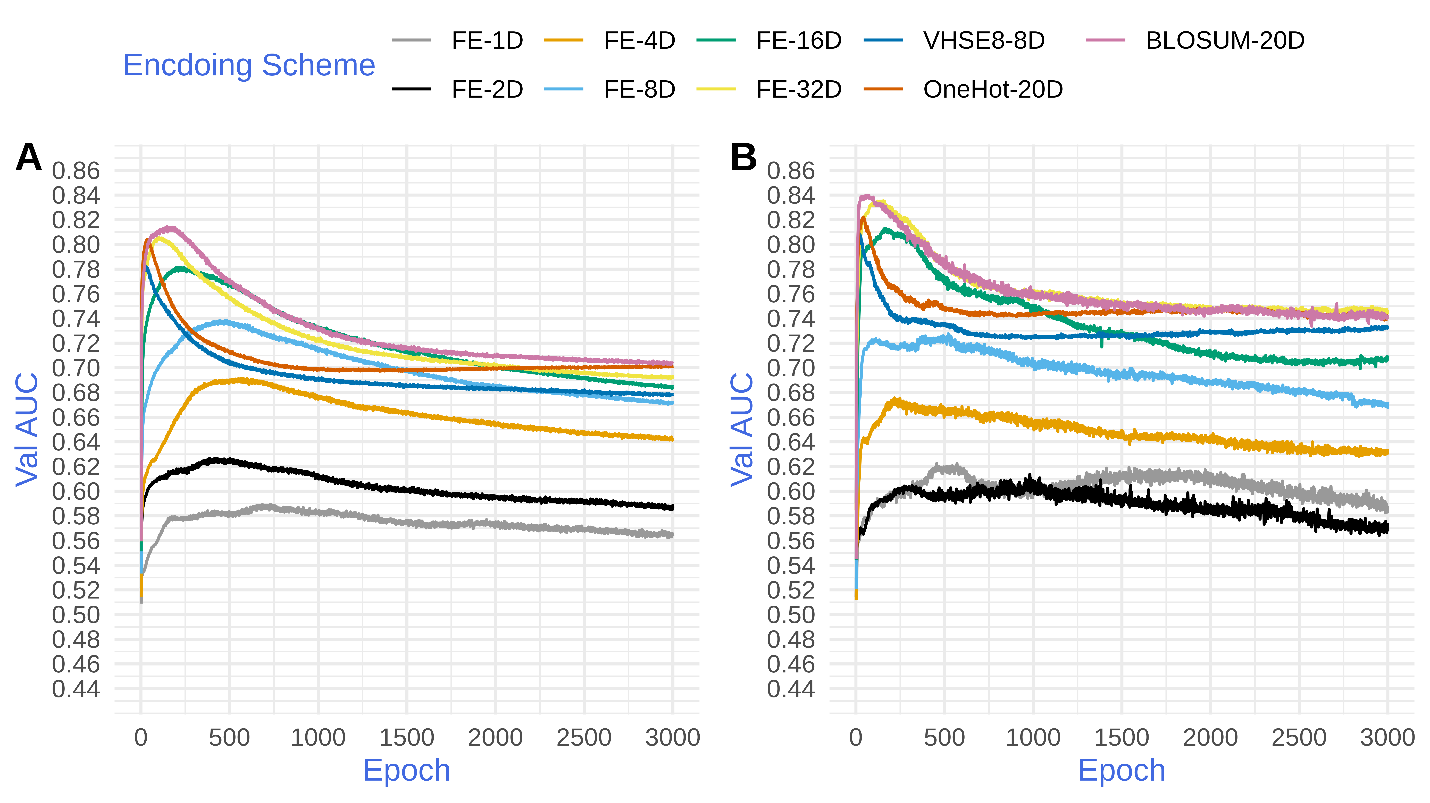

**Fig. S2**: Comparison between classical encoding and random frozen embedding (LE) encoding schemes used to encode amino acids for a CNN-LSTM based peptide-HLA-II interaction model. The y-axis shows the area under the receiver operating characteristic curve (ROC), AUC, for the model predictions on the validation dataset (Val AUC). The x-axis shows the number of training cycles or epochs. (A) shows the performances of models trained on HLA-DRB1*15:01 data and (B) shows the model performances for HLA-DRB1*13:01 data. As training deep learning models is a non-deterministic process, each line in the graph represents the average performance over three repetitions of a five-fold cross-validation dataset, i.e. fifteen different experiments.

**
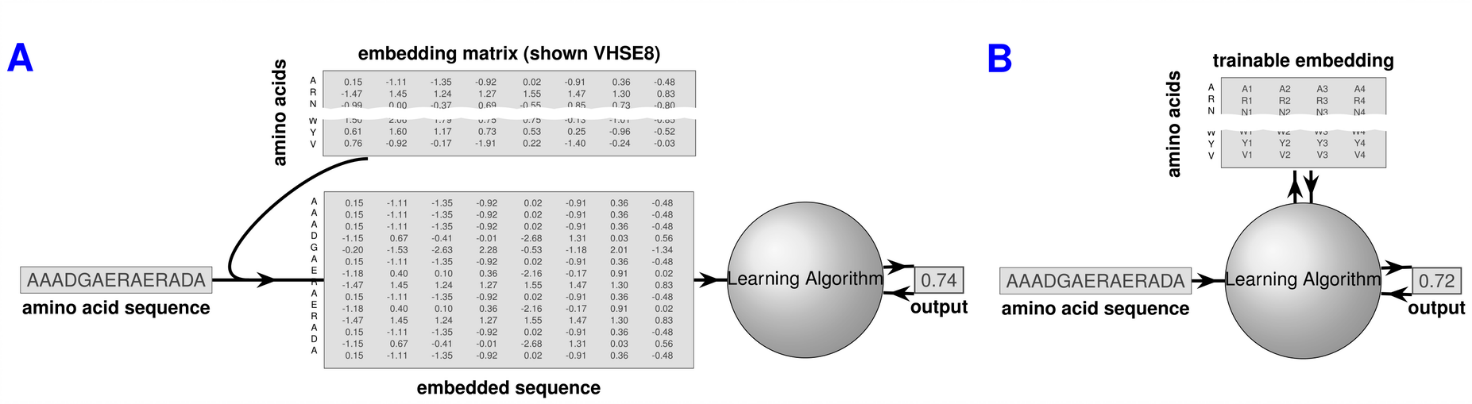
**

**Fig. S3:** Comparison between classical encoding schemes and machine-learnt encoding schemes. In the classical encoding approach (A) each amino acid has an associated vector that is predefined and the task of the network is to solve the problem using this representation. (B) In the machine-learnt approach, the associated vectors are not-predefined and the algorithm iteratively updates them to minimize the error.

**Table S1:** The parameters of the four convolution modules used with the model. For convolutional module 1-3 average pooling was used and for convolutional module four global average pooling was used.

| **Convolutional module Index** | **Number of filters** | **Kernel size** | **Stride size** |
| --- | --- | --- | --- |
| 1 | 64 | 5 | 1 |
| 2 | 128 | 7 | 1 |
| 3 | 256 | 9 | 1 |
| 4 | 512 | 15 | 1 |
